# Dogs with divergent serology for visceral leishmaniasis as sources of *Leishmania* infection for *Lutzomyia longipalpis* phlebotomine sand flies – an observational study in an endemic area in Brazil

**DOI:** 10.1371/journal.pntd.0008079

**Published:** 2020-02-20

**Authors:** Marília Fonseca Rocha, Érika Monteiro Michalsky, Fabiana de Oliveira Lara-Silva, Josiane Lopes Valadão, João Carlos França-Silva, Letícia Cavalari Pinheiro, Joel Fontes de Sousa, Ronaldo Cardoso dos Santos, Marcelo Dias Soares, Consuelo Latorre Fortes-Dias, Edelberto Santos Dias

**Affiliations:** 1 Instituto René Rachou/FIOCRUZ, Belo Horizonte, Minas Gerais, Brazil; 2 Universidade Estadual de Montes Claros, Montes Claros, Minas Gerais, Brazil; 3 Secretaria Municipal de Saúde de Montes Claros, Montes Claros, Minas Gerais, Brazil; 4 Universidade Federal de Minas Gerais, Belo Horizonte, Minas Gerais, Brazil; 5 Fundação Ezequiel Dias, Belo Horizonte, Minas Gerais, Brazil; National Institutes of Health, UNITED STATES

## Abstract

Visceral leishmaniasis (VL) is a neglected tropical disease, caused by *Leishmania* (Kinetoplastida, Trypanosomatidae) species. In Brazil, the transmission of this parasite essentially occurs through the bite of *Lutzomyia longipalpis* (Diptera: Psychodidae: Phlebotominae) previously infected with *Leishmania infantum*. Aiming at preventing VL expansion over the country, integrated control actions have been implemented through a Visceral Leishmaniasis Surveillance and Control Program (VLSCP). Among the actions currently adopted by the program, the screening-culling of seropositive dogs for canine VL (CVL) is particularly polemic. Dogs with negative or divergent serology for CVL remain in their owner’s domicile and are monitored by public health agents. In the present study, we determined the prevalence of CVL and analyzed the implementation of the VLSCP screening-culling action, in an area in Brazil where there has been a recent expansion of VL. Canine census surveys were conducted semiannually for two years (Aug/2015 to Feb/2017). Serological diagnosis of CVL was performed in accordance with current VLSCP protocol: immunochromatography (TR-DPP) followed by enzyme-linked immunosorbent assay (ELISA EIE). 6,667 dogs were serologically screened for CVL, of which 567 (8.5%) were positive in both tests and 641 (9.6%) had divergent results. A variable percentage (6.3% to 65.4%) of the dogs in the latter group became positive within nine months from the first result. Xenodiagnosis was conducted in canine samples belonging to any of the three possible serological statuses for CVL–positive, divergent or negative. *Leishmania* spp. DNA was detected in *Lu*. *longipalpis* that fed on 50.0% (5/10) of dogs with positive serology and on 29.4% (5/17) of dogs with divergent serological status for CVL. Therefore, dogs with divergent serology for CVL may be as *Leishmania*-infective to *Lu*. *longipalpis* as seropositive ones. Even with the adoption of euthanasia for seropositive dogs, part of the canine population will continue to serve as a source of *Leishmania* infection for phlebotomine sand flies.

## Author summary

Visceral leishmaniasis (VL) is a neglected tropical disease caused by a protozoan of the genus *Leishmania*. In Brazil, the parasites are mainly transmitted through the bite of female *Lutzomyia longipalpis* sand flies that have been previously infected with *Leishmania infantum*. Dogs are the main domestic reservoirs of *Leishmania*. The Brazilian Ministry of Health has implemented euthanasia of VL-seropositive dogs as a measure to prevent VL expansion in the country, using the Dual-Path Platform Immunochromatographic Rapid Test (TR-DPP) as the screening method and the enzyme-linked immunoassay (ELISA EIE) as the confirmatory test for diagnosis. Dogs with positive results in both tests are considered seropositive and recommended for euthanasia. Dogs with divergent serology between these tests remain at their owners’ home and part of them become seropositive within months. Therefore, even adopting euthanasia of seropositive dogs, part of the canine population will continue to serve as a source of *Leishmania* infection for sand flies, causing new canine and / or human cases of the disease. Difficulties and limitations to implement the screening-culling policy are also discussed.

## Introduction

Visceral leishmaniasis (VL) is a neglected tropical disease caused by species of the genus *Leishmania* belonging to the *donovani* complex. In Brazil, VL occurs in 23 of the 27 states [1]. The etiological agent of VL is *Leishmania (Leishmania) infantum* (Nicolle, 1908) (Kinetoplastida, Trypanosomatidae) [2, 3]. Parasite transmission to man and other mammalian hosts essentially occurs through the bite of previously-infected females of phlebotomine sand flies (Diptera: Psychodidae: Phlebotominae) [4]. In Brazil, the main vector of *Le*. *(Le*.*) infantum* is *Lutzomyia (Lutzomyia) longipalpis* (Lutz & Neiva, 1912).

In the epidemiological cycle of VL, dogs (*Canis familiaris*) are the most important domestic reservoirs of *Le*. *infantum* [5, 6]. The high prevalence of canine infection in endemic areas, the intense cutaneous parasitism presented by infected animals, and their close coexistence with humans reinforce this point [7–9]. Aiming at preventing VL spread over the country, the Brazilian Ministry of Health has adopted integrated actions, through the Visceral Leishmaniasis Surveillance and Control Program (VLSCP). According to the average number of human cases (n) reported by public health services in the last three years, there are three levels of epidemiological transmission risks (ETRs) attributed to Brazilian areas: sporadic (n < 2.4), moderate (2.4 ≤ n < 4.4) or intense (n ≥ 4.4). Control actions are prioritized where ETRs are intense or moderate, considering the existing data [10].

VLSCP is based on five basic control measures: early diagnosis and treatment of confirmed human cases; diagnosis and euthanasia of seropositive dogs; use of residual insecticides to control vector density; rigorous epidemiological surveillance, and health education. In 2006, the Health Surveillance Secretariat of the Ministry of Health included environmental management as a complementary action for vector control [10]. Among the current VL control measures, the effectiveness of screening-culling of seropositive dogs has been mostly questioned [3, 11–13]. Screening-culling recommendation is based on the fact that positive animals serve as sources of infection for phlebotomine sand fly vectors, which, in turn, transmit the parasite to other mammals, including dogs and humans [14, 15]. Presently, canine VL (CVL) diagnosis is performed through a screening method using rapid immunochromatography Dual-Path Platform (TR-DPP) followed by enzyme-linked immunosorbent assay (ELISA EIE) for confirmation. Dogs that are reactive in both tests are considered serologically positive and are taken to euthanasia, in accordance with VLSCP policy. Those with negative or divergent serology remain in the owners’ domiciles.

In the present study we analyzed data from a Brazilian area that used to show sporadic ETR for VL until 2014, with no human cases in 2012, three cases in 2013 and three in 2014. But, in the following year (2015), two new cases were registered by the Public Health Service and the ETR switched to moderate for the triennium 2013 to 2015, which led to the systematic application of VLSCP control measures. We followed the screening-culling policy from Aug/2015 to Feb/2017 and evaluated: (1) the impact of this strategy on CVL prevalence in the studied area; (2) the reliability of the serological tests currently employed for CVL diagnosis; (3) alterations in the serological statuses of divergent dogs after the first diagnosis result; (4) *Leishmania* infectivity of divergent dogs to *Lu*. *longipalpis*.

## Materials and methods

### Study area

**Fig 1:**
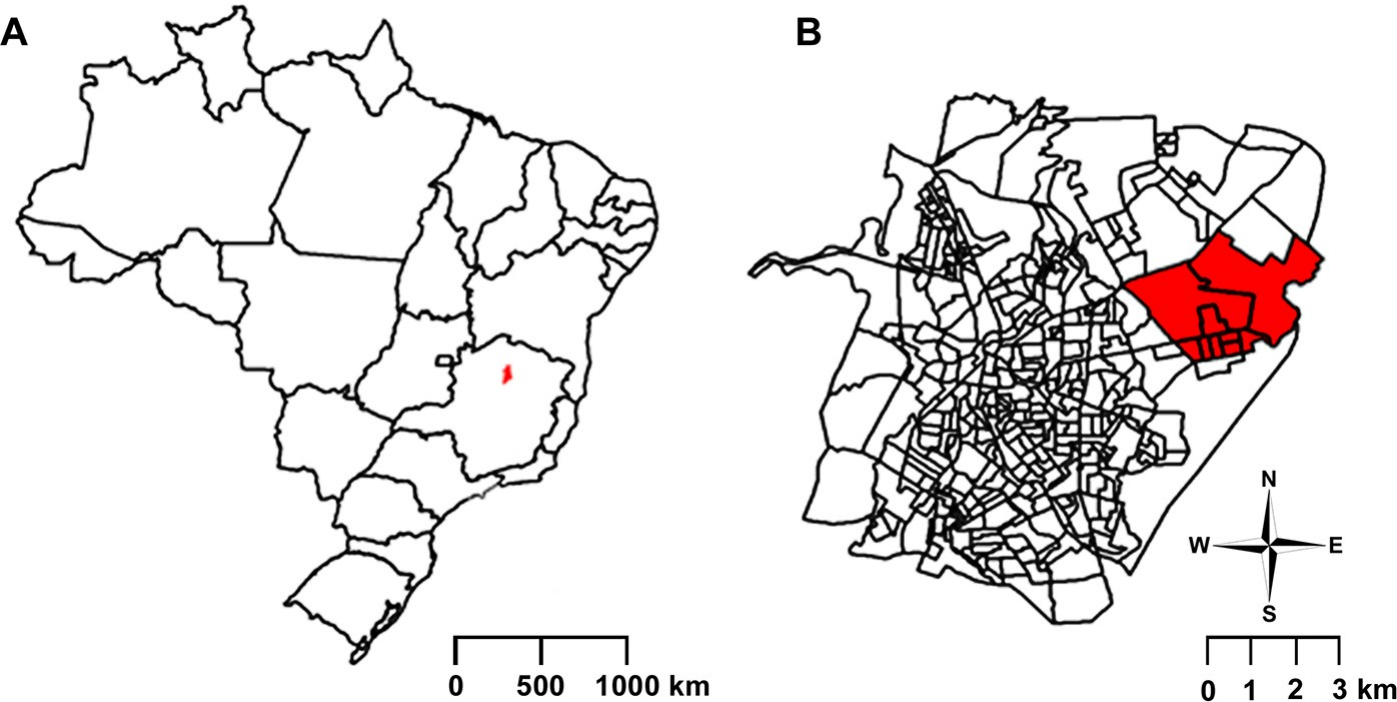
A. Geographic localization of Montes Claros city (in red) in Minas Gerais state, Brazil; B. Geographic localization of Independência neighborhood (in red) in Montes Claros, Minas Gerais, Brazil. Created by the authors using Adobe Photoshop CS4 software.

The municipality of Montes Claros (16^o^43`41"S, 43^o^51`54" W) is located 420 km north from the capital of Minas Gerais state, in Brazil (Fig 1). It occupies approximately 3,569 km^2^, being 39 km^2^ of urban area and 3,530 km^2^ of rural zone. It is the sixth most populous municipality in the state, with 404,804 inhabitants [16]. Montes Claros has important universities and industries, being a cultural and regional reference in the north of Minas Gerais. Its climate is tropical semi-humid (hot and dry), with an average temperature of around 25°C. For VLSCP purposes, the city was subdivided into nineteen sectors and specific ETRs were calculated for each sector. Our study was performed in Independência neighborhood, located in the 16^th^ sector of Montes Claros (Fig 1). Half of the VL cases (4/8) reported in the triennium 2013–2015 occurred in this neighborhood, which comprises 6,196 properties distributed in 190 blocks, with an estimated population of 11,319 inhabitants.

### CVL diagnosis

Two sequential serological techniques were used for CVL diagnosis, as recommended by VLSCP [17]. The Rapid Dual Path Platform (TR-DPP-LVC, Bio-Manguinhos, Rio de Janeiro) was performed as a screening test. This is a qualitative test for the detection of antibodies anti-*Leishmania* of the *Le*. *donovani* complex using the recombinant protein K28 (fragments K26, K39 and K9) as antigen. The dogs that were reactive in this test had their serum tested by enzyme linked immunosorbent assay (EIE visceral canine leishmaniasis, Bio-Manguinhos, Rio de Janeiro), which quantifies sera or plasma antibodies that recognize *Leishmania major*-like (strain MHOM/BR/71/BH121) antigens purified from *in vitro* cultures. Samples with optical densities higher than the cut-off value of 20% were classified in the grey zone of the assay. The results of these serological tests defined three serological statuses for CVL: positive (positive DPP and positive ELISA), negative (negative DPP and negative ELISA), or divergent (positive DPP, and negative or grey zone ELISA).

### Canine sample

All dogs domiciled in Independência neighborhood were tested for CVL in four canine census surveys (CCSs) conducted every six months for two years: Aug/2015 (CCS1), Feb/2016 (CCS2), Aug/2016 (CCS3) and Feb/2017 (CCS4). Each survey included the current canine population at that time, but not necessarily the same dogs, due to euthanasia of reactive dogs, refusal of the dog’s owner to participate in the canine test, or uninhabited domiciles. Animal replacements were not individually monitored.

Dogs with a positive status in any CCS were collected for euthanasia within a month between DPP testing and the owner’s consent. Dogs with negative or divergent serological statuses in any CCS remained in the owner’s domicile. Therefore, a dog with negative or divergent serological status in a CCS might have been tested and counted up to four times during the study.

A sample of dogs with divergent serology was microchipped and followed up quarterly, for up to nine months, in order to evaluate any change in the serological status over time. Dogs that positivized were collected for euthanasia. A logistic limitation prevented the sixth and ninth follow-ups of divergent dogs from CCS4.

### Xenodiagnosis

**Fig 2:**
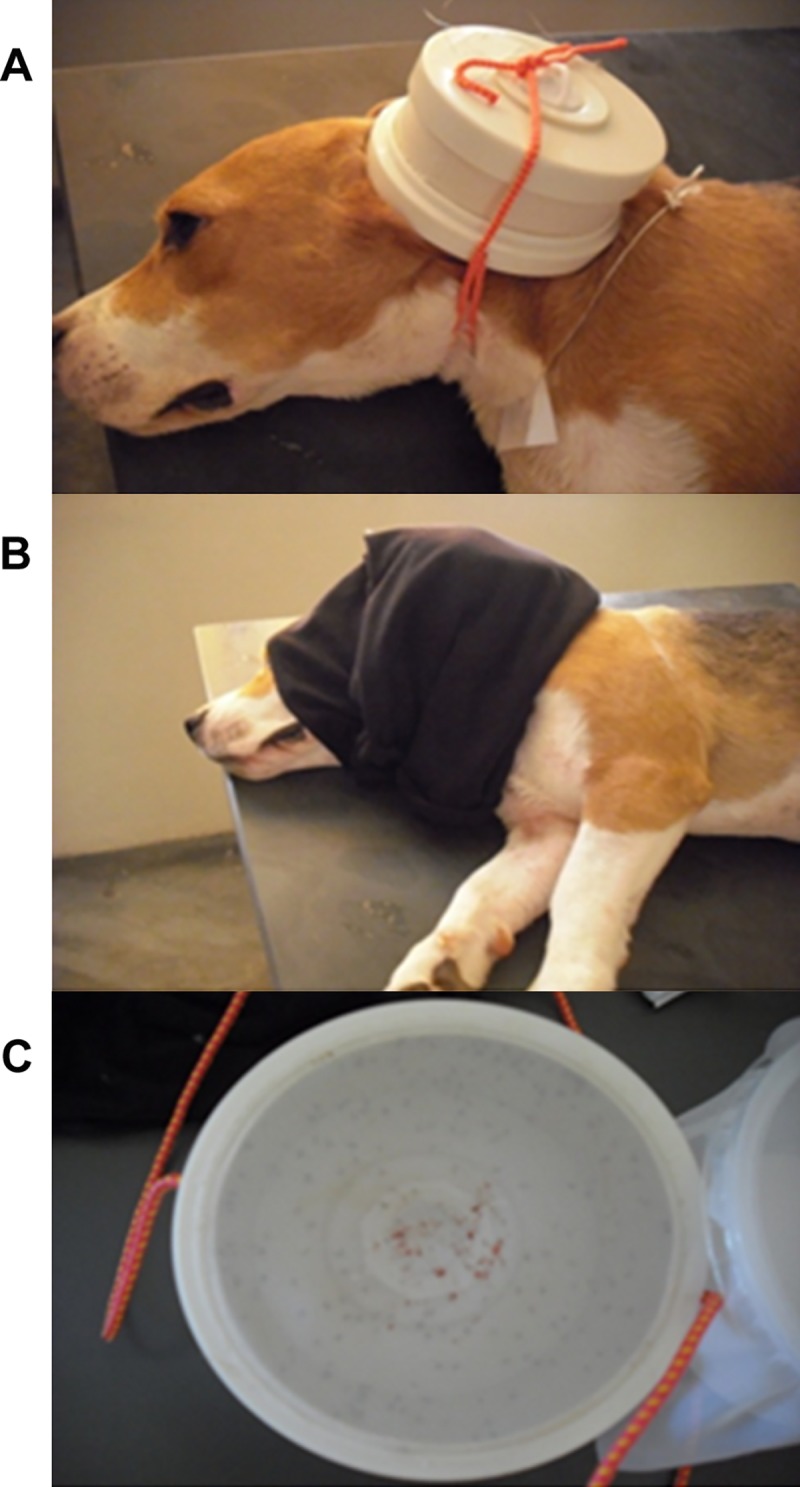
Experimental dog during xenodiagnosis. A. *Lu*. *longipalpis* females were placed in a plastic box with an open top covered by a fine nylon mesh and the box was fixed on the inner skin of the dog's ear. B. The whole set was covered with a piece of black tissue for 30 minutes. C. *Lu*. *longipalpis* specimens were collected for molecular analysis. Engorged females can be noticed near the nylon mesh.

A group of divergent dogs from CCS4 (Feb/2017) was submitted to xenodiagnosis (Fig 2) to assess *Leishmania* infectivity for *Lu*. *longipalpis*. Dogs with positive and negative serological statuses were included as controls. Blood samples were named “blood sample A”. The sample size in each serological group was defined by convenience. Inclusion criteria were the serological status in CCS4 and the owner’s consent by signing the Informed Consent Statement. Exclusion criteria were aggressive behavior, co-morbidities that contraindicated the anesthetic procedure, large sized dogs, dogs over 10 years of age, pregnant female dogs, or dogs in the proestrus or estrus phases of the reproductive cycle. Immediately before the xenodiagnosis procedure (March 2017), fresh blood samples (named “blood sample B”) were collected from every dog for new CVL diagnosis tests.

Dogs were sedated with xylazine and ketamine solution, which was administered according to the body weight of the animal. Thirty females and five males of first generation (F1) *Lu*. *longipalpis* were placed in round plastic boxes (10 cm diameter, 5 cm high) with an open top covered by a fine nylon mesh. This top was placed on the inner skin of the dog's ear and the whole set was covered with a piece of black tissue to favor blood feeding by the phlebotomine sand flies. After 30 minutes of exposure, the specimens were transferred to breeding cages, where they were kept alive, at 25-28^o^ C and 90% relative humidity. Seven days after blood feeding, the surviving insects were sacrificed and the total DNA was extracted for molecular analysis.

### DNA extraction and specific *Leishmania* identification

*Lu*. *longipalpis* females used in xenodiagnosis were transferred to conical microtubes and sample pools were prepared per dog. Total DNA was extracted using the Puregene Core Kit A (QIAGEN). DNA extraction reliability was verified by the amplification of a 220 bp fragment corresponding to the IVS6 region of a constitutive gene (cacophony) from *Lutzomyia* phlebotomine sand flies [18]. The presence of *Leishmania* DNA in total DNA was investigated using *Leishmania*-nested PCR (LnPCR) for the small subunit ribosomal RNA (SSUrRNA) gene [19, 20]. In the first amplification step, ten to twenty ng of extracted DNA were amplified in the presence of Kinetoplastida-specific primers: R221 (^5’^GGTTCCTTTCCTGATTTACG^3’^) and R332 (^5’^GGCCGGTAAAGGCCGAATAG^3’^). Positive samples for *Leishmania* showed a conserved 603 bp fragment. The PCR product was then diluted 1:40 in sterile water and used as template in the second amplification step with *Leishmania*-specific primers: R223 (^5’^TCCCATCGCAACCTCGGTT^3’^) and R333 (^5’^AAAGCGGGCGCGGTGCTG^3’^). Positive samples generated a 358 bp fragment. LnPCR cycling conditions were those previously described [18, 20]. The amplified products were visualized under UV light, after electrophoresis on 2% agarose and ethidium bromide staining. Negative (no DNA) and positive (20 ng of DNA extracted from *Leishmania chagasi*—MHOM/BR74/PP75) were run as controls. For all PCR amplifications, we used PureTaq Ready-To-Go PCR Beads (GE Healthcare) in a Veriti 96 well Thermo Cycler (Applied Biosystems).

### Statistical analysis

Xenodiagnosis results for dogs with positive and divergent serological statuses were compared by Fisher’s exact test. The analyses were performed using Prism 6 (GraphPad Inc., EUA) with 95% of confidence.

### Ethical statement

All procedures were conducted in accordance with the guidelines of the Brazilian Animal Experimental College (Law no. 11794) and followed the technical norms established by the Federal Board of Veterinary Medicine (Resolution no. 1000/2012). The study protocols were approved by the Animal Research Ethics Committee of Fundação Oswaldo Cruz (CEUA/FIOCRUZ) under number LW-15/16. The dogs’ owners were informed as to the objectives of the xenodiagnosis and voluntarily signed the Informed Consent Statement.

## Results

A total of 6,667 dogs were tested for CVL in semiannual surveys (CCS1 to CCS4) during two years. Positivity rates varied between 6.1% to 9.6%, depending on the CCS. At the end of the study, 567 dogs (8.5%) were positive for the disease (Table 1). The great majority of them (455 animals or 80.2%) were euthanized as determined by VLSCP. The reasons for non-accomplishment of 100% of the recommended euthanasia are listed in Table 2.

**Table 1:** Canine census surveys for canine visceral leishmaniasis performed semiannually in an area of recent moderate epidemiological risk of transmission of visceral leishmaniasis. The study was performed in Independência neighborhood in Montes Claros (Minas Gerais state, Brazil). Aug/2015 to Feb/2017.

Canine census	Date	No. dogs		Positivity
survey (CCS)	(Month/year)	Tested	Positive	(%)
1	Aug/2015	1693	163	9.6
2	Feb/2016	1833	112	6.1
3	Aug/2016	1630	156	9.6
4	Feb/2017	1511	136	9.0
Total	-	6667	567	8.5

**Table 2:** Seropositive dogs recommended to euthanasia, in accordance with the Visceral Leishmaniasis Surveillance and Control Program (VLSCP) guidelines of the Brazilian Ministry of Health. The study was performed in an area of recent moderate epidemiological risk of transmission (ETR). Montes Claros (Minas Gerais state, Brazil). Aug/2015 to Feb/2017.

Canine census survey	No. seropositive dogs	Euthanasia				Reason for no euthanasia							
		Yes		No		Owner’s refusal		Death		Address change		Unknown	
		No. dogs	%	No. dogs	%	No.	%	No. dogs	%	No. dogs	%	No. dogs	%
1	163	135	82.8	28	17.2	9	32.1	11	39.3	8	28.6	0	0
2	112	92	82.1	20	17.9	10	50.0	8	40.0	2	10.0	0	0
3	156	132	84.6	24	15.4	15	62.5	7	29.2	1	4.2	1	4.2
4	136	96	70.6	40	29.4	26	65.0	7	17.5	4	10.0	3	7.5
Total	567	455	-	112	-	60	-	33	-	15	-	4	-
Mean±S.D.	-	-	80.0±6.4	-	20.0±6.4	-	52.4±15.0	-	31.4±10.5	-	13.2±10.6	-	2.9 ±3.6

Six hundred and forty-one dogs (9.6% of the total tested) displayed a divergent serological status for CVL in the first CVL testing, considering the four surveys. A percentage of this specific population was serologically accompanied for up to nine months (Table 3). It is worthy to note that, three months after the first testing, 74.4% (477 dogs) of the animals with divergent results became positive, with conversion rates of up to 63.6% (CCS2). Lower seroconversion rates were observed after six months (11.5%) compared to three months (29.4%). The highest seroconversion rate occurred after nine months in CCS1 (65.4%), with 51 dogs positive for CVL among the 78 previously divergent ones.

**Table 3:** Follow-up of dogs with divergent serological statuses for canine visceral leishmaniasis (CVL) in an area of recent moderate epidemiological risk of transmission (ETR). New serological tests (DPP and ELISA) were performed every three months after the first diagnosis, during up to nine months. Montes Claros (Minas Gerais state, Brazil). Aug/2015 to Feb/2017.

CSS	Dogs with a divergent status at the time of CCS (no.)	Follow-ups								
		Three months			Six months			Nine months		
		Number of dogs tested	Dogs with a positive status		Number of dogs tested	Dogs with a positive status		Number of dogs tested	Dogs with a positive status	
			No.	%		No.	%		No.	%
1	184	125	25	20.0	43	7	16.3	78	51	65.4
2	95	77	49	63.6	27	2	7.4	24	3	12.5
3	203	168	54	32.1	104	11	10.6	80	5	6.3
4	159	107	12	11.2	-	-	-	-	-	-
1 to 4	641	477	140	29.4	174	20	11.5	182	59	32.4

*Leishmania* infectivity to VL vectors of dogs with positive, divergent or negative statuses for CVL was evaluated by xenodiagnosis. The number of surviving *Lu*. *longipalpis* females pooled for DNA extraction and investigation for *Leishmania* DNA varied from 10 to 28 (median = 20 specimens; 25% percentile = 15 specimens; 75% percentile = 23 specimens), depending on the dog. Table 4 summarizes the results for each canine group. Dogs were pooled for xenodiagnosis according to their serological results in CCS4 (blood sample A), being ten dogs with a positive serological status, seventeen with a divergent serological status and seven with a negative serological status. *Leishmania* spp. DNA was detected in five positive dogs and five divergent dogs, while no DNA was detected in negative dogs. There was no significant statistical difference in *Leishmania* infectivity for *Lu*. *longipalpis* between positive and divergent dogs (p-value = 0.4153). Although we used a small number of dogs in each serological group, our data suggest that divergent dogs are as *Leishmania*-infective to *Lu*. *longipalpis* as positive ones.

**Table 4:** Xenodiagnosis results of dogs serologically tested for canine visceral leishmaniasis in Montes Claros (Minas Gerais state, Brazil) according to the current protocol adopted by the Brazilian Ministry of Health. Dogs’ IDs are represented by the last five digits of the microchip. Abbreviation: gz = grey zone in ELISA (see Material and Methods). Blood sample collection: A. CCS4 (Feb/2017); B. Immediately before xenodiagnosis (Mar/2017). Study period: Aug/2015 to Feb/2017.

Dog ID	Serological diagnosis			Xenodiagnosis	Serological diagnosis		
	Blood sample A		Status	*Leishmania* DNA in *Lu*. *longipalpis*	Blood sample B		Status
	DPP	ELISA			DPP	ELISA	
85612	+	+	Positive	+	+	+	Positive
85564	+	+	Positive	+	+	+	Positive
85377	+	+	Positive	+	+	+	Positive
85628	+	+	Positive	+	+	+	Positive
85553	+	+	Positive	+	+	+	Positive
85603	+	+	Positive	-	+	+	Positive
85604	+	+	Positive	-	+	+	Positive
85640	+	+	Positive	-	+	+	Positive
85571	+	+	Positive	-	+	+	Positive
85625	+	+	Positive	-	+	gz	Divergent
80080	+	-	Divergent	+	+	+	Positive
80134	+	-	Divergent	+	+	-	Divergent
85582	+	-	Divergent	+	+	-	Divergent
79958	+	-	Divergent	+	+	-	Divergent
79976	+	-	Divergent	+	+	+	Positive
79964	+	-	Divergent	-	+	+	Positive
85368	+	-	Divergent	-	+	gz	Divergent
85443	+	-	Divergent	-	+	-	Divergent
80053	+	-	Divergent	-	-	-	Negative
79979	+	-	Divergent	-	+	-	Divergent
80001	+	-	Divergent	-	+	-	Divergent
79982	+	-	Divergent	-	+	-	Divergent
79953	+	-	Divergent	-	-	-	Negative
85643	+	-	Divergent	-	-	-	Negative
80141	+	-	Divergent	-	+	-	Divergent
80132	+	-	Divergent	-	+	-	Divergent
80117	+	-	Divergent	-	+	-	Divergent
85414	-	-	Negative	-	-	gz	Divergent
80108	-	-	Negative	-	-	gz	Divergent
80077	-	-	Negative	-	-	-	Negative
85595	-	-	Negative	-	-	-	Negative
80032	-	-	Negative	-	+	-	Divergent
80104	-	-	Negative	-	-	-	Negative
79997	-	-	Negative	-	-	-	Negative

However, results from blood samples B did not confirm the previous serological status for CVL for every case. Among the positive dogs, one (#85625) presented divergent results in the second blood test (sample B). Among the divergent dogs, there were three positive (#80080, #79976, #79964) and three negative (#80053, #79953, ##85643) results for sample B. Three negative dogs (#85414, #80108, #80032) became divergent in the second test. If we compare only positive and divergent dogs that showed the same results in both tests, albeit taking the risk of having even smaller samples, *Leishmania* spp. DNA was detected in five among nine confirmed seropositive dogs and in three out of eleven dogs with confirmed divergent status for CVL. Confirming the previous results, divergent dogs were as *Leishmania*-infective to *Lu*. *longipalpis* as positive ones (p-value = 0.3618), in this second analysis.

## Discussion

In spite of the efforts to control VL in Brazil, the disease is still expanding throughout the country [21–26]. Among the interventions recommended in VLSCP, canine euthanasia is the least approved by the community for obvious reasons. Canine euthanasia is recommended due to the high susceptibility of dogs to infect VL vectors, their ability to spread the disease from enzootic foci, the existence of a large contingent of asymptomatic animals hosting parasites in their dermis with a potential for transmission, their closeness to humans, and the usual confirmation of a CVL case preceding a human case [27–31]. Although there are no reports on VL epidemics in Brazilian cities without the presence of infected dogs, their role in the VL transmission chain is still questioned [3, 12, 32].

In the present study we show that, after two years of intervention, with euthanasia of most seropositive dogs (80.2%), no change in the positivity rates of CVL could be observed. Our data are in accordance with those obtained by [33], in Rio de Janeiro (Brazil), although other studies have described a decrease in the incidence/prevalence of CVL after screening-culling procedures [11, 34]. In contrast, a study in an endemic area for VL in Bahia (Brazil) showed that the removal of positive dogs was insufficient to control CVL, but reduced the strength of canine infection, with a consequent decrease in VL incidence [11]. This continued VL transmission may be associated with the efficiency and time of removal of seropositive dogs or with the low acceptance of canine euthanasia by the owners, especially regarding asymptomatic animals [3, 11, 35, 36]. In fact, more than 50% of the non-euthanized dogs in the present study were due to the refusal of the dog’s owner.

It is important to notice that the canine screening-culling strategy depends directly on the quality, reliability, sensitivity and specificity of the diagnostic tests employed. In spite of some recent improvements, none of the available tests has reached 100% sensitivity and 100% specificity for CVL. Better diagnosis techniques must be developed to express the real magnitude of infection of the canine population [3, 37, 38]. Low-sensitivity tests may explain the false-negative results, which enable the maintenance of the transmission cycle. Low-specificity tests may result in the removal of false-positive, uninfected dogs, which discredits the controlling actions among the population, especially for those who have an affective bond with the animal [33, 39].

In December 2011, the Brazilian Ministry of Health replaced the diagnosis protocol of CVL (Brazilian Ministry of Health, 2011). Until then, serological screenings were performed by ELISA and the positive results were confirmed by indirect immunofluorescence assay. After 2012, the Dual-Path Platform (DPP) has been recommended as the screening test, followed by ELISA for confirmation [40]. The accuracy of these two protocols was compared using 780 serum samples randomly collected from a VL endemic area, using parasite cultures and real-time PCR as references. The current protocol presented higher specificity (0.98 vs. 0.95) and higher positive predictive value (0.83 vs. 0.70) than the previous one. The sensitivity of both protocols was similar (0.73) but a reduction in the number of false positives was observed after the change [41]. Unfortunately, alternative methods with higher sensitivity and specificity, at affordable costs for public health services, are still unavailable [20, 42]. Coinfection or cross-reaction with *Babesia canis* and *Ehrlichia canis* in dogs from urban endemic or non-endemic areas for CVL were shown to interfere with the serological diagnosis of CVL [32, 43–45]. Serological cross-reaction was also observed with *Trypanosoma cruzi*, which belongs to the same Trypanosomatidae family as *Le*. *infantum* [46]. In the present study, the highest prevalence of CVL (9.6%) was noticed in CCS1 and CCS3, which were diagnosed in August 2015 and August 2016, respectively. The period coincides with peaks of tick infestation due to the dry season (April to October). It is possible that these pathological agents, which are transmitted by ectoparasites, interfere in CVL diagnosis.

Along with the difficulty in identifying all infected dogs and interrupting the transmission cycle, immediate replacement of removed dogs by susceptible pups or by other infected dogs, and the possibility of involvement of other reservoirs in the maintenance of canine infection may compromise the efficacy of the screening-culling strategy for CVL control [3, 47–49]. Moreover, this preventive action is operationally compromised by difficulties in implementation and costs, heterogeneity of disease transmission within the assessed areas, limited number of clusters for comparison, and the complexity of random allocations due the mobility of the canine population [3, 31, 47, 48].

The various difficulties in performing the actions determined by VLSCP have been recently evaluated through interviews with six coordinators from important Brazilian municipalities with canine and/or human transmission (Campinas, Bauru, Goiânia, Campo Grande, Fortaleza, and Belo Horizonte). Besides the resistance of dogs’ owners to euthanasia, two other problems have been reported: discontinuity of control measures and low coverage of chemical controls. The authors suggested the need to review VLSCP, given the impossibility of fully complying with its guidelines at the municipal level [50].

The main limitation of the present study was the small sample size for xenodiagnosis, which became even smaller when a confirmation of the serological canine status immediately before xenodiagnosis was included as a requirement. Among the drawbacks to overcome this limitation, we faced difficulties in convincing the dogs’ owners to authorize their participation, especially for seronegative or divergent dogs, because they would have to be removed from their domicile, with inherent risks of anesthesia for the experimental procedure. Moreover, some owners showed little trust in the public health service and some received contrary orientations from non-governmental organizations for animal protection or private veterinary physicians. On the other hand, seropositive dogs had to be removed from the domicile to the public kennel to be euthanized, as soon as possible.

Nevertheless, our study shows that the screening-culling strategy had no impact on CVL prevalence in the studied area, even under optimized conditions such as semiannual canine census surveys and a faster removal of reactive dogs for euthanasia. In a very recent study, [51] reviewed the failures and lack of efficacy of the culling policy in reducing leishmaniasis incidence in countries where it has been adopted. According to the authors, “unless dogs are screened (and eventually eliminated) monthly, there will always be newly infected dogs if sand flies and other animal reservoirs are present”.

It is noteworthy that, in our study, 74.4% (477) divergent dogs seroconverted to positive three months after the first CVL test, in all four CCSs (Table 4). We found no reason for the lower seroconversion rates observed six months after the CCSs. The highest seroconversion rate (65.4%), which occurred nine months after CCS1, may be explained by the intensification of dialogue with dog owners, associated to the appearance CVL-suggestive clinical signs. Most importantly, our data indicate that divergent dogs may be as *Leishmania*-infective to *Lu*. *longipalpis* as seropositive ones, but the hypothesis of seroconversion due to new *Leishmania* infections cannot be discarded, since they remained in the area of transmission.

In conclusion, we suggest the serological follow-up of divergent dogs to be included in the control measures adopted by VLSCP, as well as diagnosis test repetitions within the operational capacity of the Public Health Services. It is also important to advise dog owners to seek diagnosis tests, if the animal shows clinical signs compatible with the disease. Whenever possible, individual preventive measures, such as collars impregnated with insecticides or topical repellent substances, should be adopted.
